# Case report: Homozygous variants of *NEB* and *KLHL40* in two Arab patients with nemaline myopathy

**DOI:** 10.3389/fgene.2023.1098102

**Published:** 2023-03-21

**Authors:** Cristina Skrypnyk, Aseel Ahmed Husain, Hisham Y. Hassan, Jameel Ahmed, Abdulla Darwish, Latifa Almusalam, Noureddine Ben Khalaf, Fahad Al Qashar

**Affiliations:** ^1^ Department of Molecular Medicine, Al‐Jawhara Centre for Molecular Medicine, Arabian Gulf University, Manama, Bahrain; ^2^ Department of Medical Genetics, University Medical Center, King Abdulla Medical City, Manama, Bahrain; ^3^ Department of Pediatrics, Bahrain Defence Force Hospital, Royal Medical Services, Riffa, Bahrain; ^4^ Banoon ART and Cytogenetics Centre, Bahrain Defence Force Hospital, Royal Medical Services, Riffa, Bahrain; ^5^ Radiology Department, University Medical Center, King Abdulla Medical City, Manama, Bahrain; ^6^ Department of Pathology, Bahrain Defence Force Hospital, Royal Medical Services, Riffa, Bahrain; ^7^ Life Sciences Department, Health Biotechnology Program, College of Graduate Studies, Arabian Gulf University, Manama, Bahrain

**Keywords:** congenital, myopathy, nemaline, WES, NEB, KLHL40

## Abstract

**Objective:** Nemaline myopathies are a heterogeneous group of congenital myopathies caused by mutations in different genes associated with the structural and functional proteins of thin muscular filaments. Most patients have congenital onset characterized by hypotonia, respiratory issues, and abnormal deep tendon reflexes, which is a phenotype encountered in a wide spectrum of neuromuscular disorders. Whole-exome sequencing (WES) contributes to a faster diagnosis and facilitates genetic counseling.

**Methods:** Here, we report on two Arab patients from consanguineous families diagnosed with nemaline myopathy of different phenotype spectrum severities.

**Results:** Clinical assessment and particular prenatal history raised suspicion of neuromuscular disease. WES identified homozygous variants in *NEB* and *KLHL40*. Muscle biopsy and muscle magnetic resonance imaging studies linked the genetic testing results to the clinical phenotype. The novel variant in the *NEB* gene resulted in a classical type 2 nemaline myopathy, while the *KLHL40* gene variant led to a severe phenotype of nemaline myopathy, type 8. Both patients were identified as having other gene variants with uncertain roles in their complex phenotypes.

**Conclusions:** This study enriches the phenotypic spectrum of nemaline myopathy caused by *NEB* and *KLHL40* variants and highlights the importance of detailed prenatal, neonatal, and infancy assessments of muscular weakness associated with complex systemic features. Variants of uncertain significance in genes associated with nemaline myopathy may be correlated with the phenotype. Early, multidisciplinary intervention can improve the outcome in patients with mild forms of nemaline myopathies. WES is essential for clarifying complex clinical phenotypes encountered in patients from consanguineous families. Targeted carrier screening of extended family members would enable accurate genetic counseling and potential genetic prevention.

## Introduction

Nemaline myopathies (NMs) are a heterogeneous group of congenital myopathies caused by mutations in different genes associated with the structural and functional proteins of thin muscular filaments. The incidence of NM is estimated to be 1 in 30,000 births, with a carrier frequency of approximately 1/87 (Lehtokari et al., 2014). The spectrum of clinical phenotypes is very wide, ranging from severe neonatal forms to mild disorders in childhood (Sewry et al., 2019; Wang et al., 2020; Wen et al., 2020). NMs commonly show slow or no progression of symptoms, and only 16% of severe neonatal cases present with hypotonia, arthrogryposis, cardiomyopathy, and respiratory failure leading to early infant death (Cassandrini et al., 2017). Generalized weakness is part of the pediatric clinical phenotype of a wide spectrum of neuromuscular disorders and poses clinical diagnostic challenges. Whole-exome sequencing (WES) is now replacing more invasive procedures such as muscle and nerve biopsies and is helping clinicians reach a diagnosis faster (Mubaraki, 2021).

Nebulin is a giant protein of the skeletal sarcomeres that accounts for 4% of the total myofibrillar protein. Homozygous or compound heterozygous pathogenic variants in the nebulin gene (*NEB*) cause autosomal recessive nemaline myopathy type 2 (NEM2) (OMIM # 256030), which accounts for more than 50% of all NMs (Jin et al., 2014; Malfatti et al., 2014; Piga et al., 2016; Laitila et al., 2020; Laflamme et al., 2021). NEM2 appears in infancy with hypotonia and muscle weakness affecting the face, neck flexors, and proximal limb muscles. The most severe forms of NEM2 lead to death in the first 2 years of life (North and Ryan, 2002).

Nebulin and leiomodin 3, as striated skeletal muscle thin filaments, are stabilized by a kelch-like family member 40 protein detected in striated human fetal muscles, with the highest expression in adult skeletal muscles (Laitila and Wallgren-Pettersson, 2021). This protein maintains sarcomere integrity by modulating the regulatory circuits that control the expression of muscle-specific proteins (Cenik et al., 2015). Sarcomere dysfunction can cause secondary changes in the muscle’s ultrastructure, such as the nemaline rods, that further worsen muscle weaknesses (de Winter and Ottenheijm, 2017). Homozygous or compound heterozygous mutations in the kelch-like family member 40 gene (*KLHL40*) cause severe autosomal recessive nemaline myopathy type 8 (NEM8) (OMIM # 615348). Deficiencies in *KLHL40* gene expression lead to marked reductions in nebulin and leiomodin 3 proteins, structural sarcomere irregularities, and a lethal loss of muscle function (Ravenscroft et al., 2013; Garg et al., 2014). KLHL40 variants are associated with severe forms of NMs that present with fetal akinesia or hypokinesia, contractures, fractures, respiratory failure, swallowing difficulties at birth, and early death in infancy (Kawase et al., 2015).

## Methods

We report two rare cases of nemaline myopathy with different clinical severity spectrums caused by homozygous variants in *NEB* and *KLHL40*, in two consanguineous Arab families (ClinVar accessions SCV003762114--SCV003762117). Clinical and laboratory data were collected from their medical records and analyzed. An extended literature review of previously reported cases with *NEB* and *KLHL40* gene variants was conducted. The research protocol was approved by the appropriate research ethics committees (approval numbers: E016-PI-6/21 and BDF/R&REC/2022-659), and written consent for the publication of both cases, including the use of photographs, was obtained from the parents.

## Results

### Case 1

**FIGURE 1:**
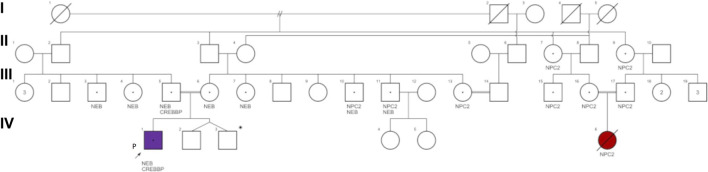
Family pedigree of patient 1. NEB-nebulin gene variant c. 11969A>C; CREBBP: cyclic adenosine monophosphate response element binding protein gene variant c.5600G>A; NPC2: NPC intracellular cholesterol transporter 2 gene variant c.420-422delCTG. Proband (P) IV1 (purple): patient 1 was diagnosed with nemaline myopathy type 2 (NEB gene homozygous c.11969A>C) and heterozygous for CREBBP variant c.5600G>A; IV6 (red) diagnosed with Niemann-Pick disease type C2 (NPC2 gene homozygous c.420-422delCTG). (IV-2 & IV-3)* are dizygotic twins through pre-implantation genetic testing for monogenic disorders (PGT-M).

**FIGURE 2:**
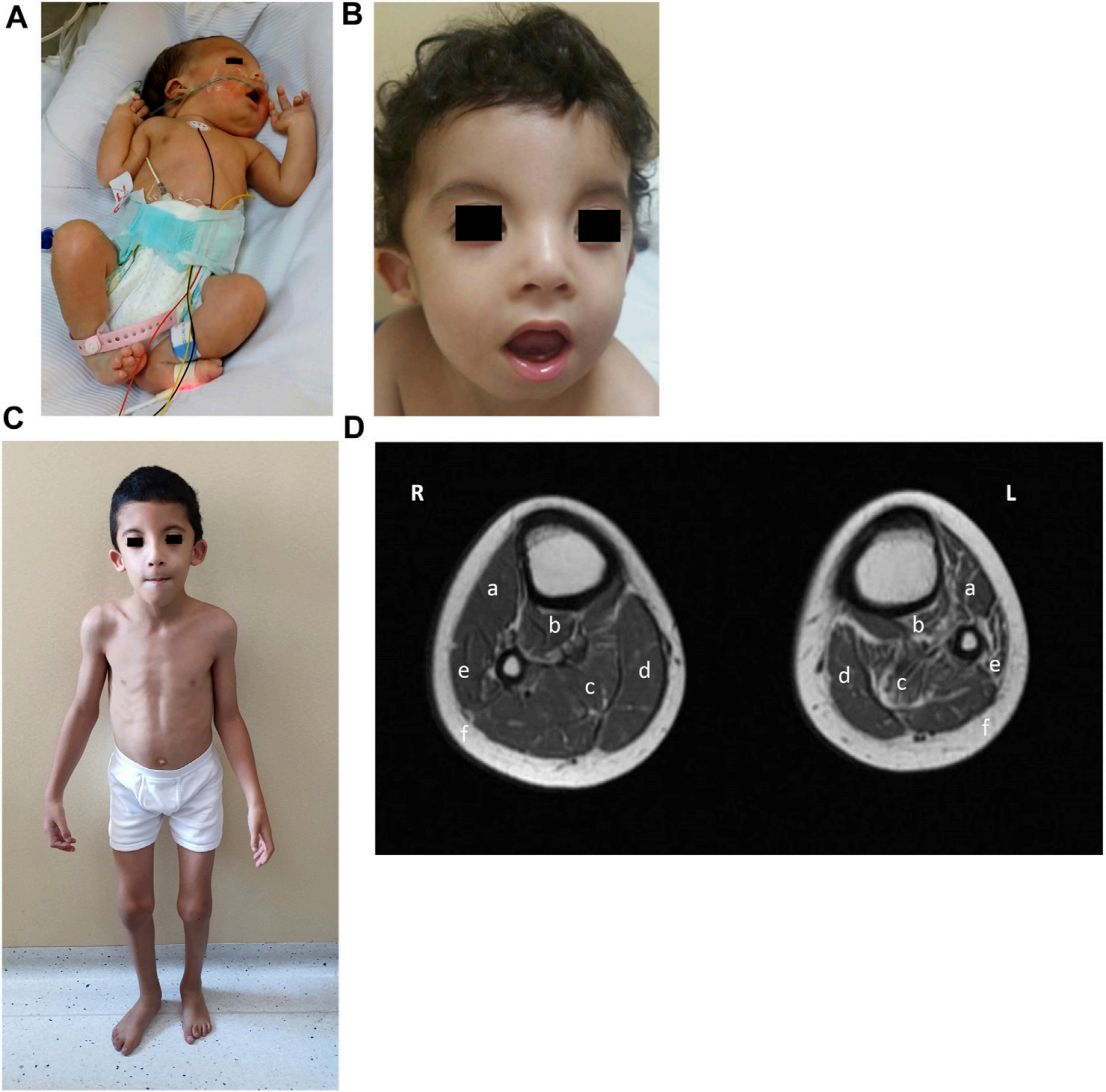
Phenotype of patient 1. **(A)** Clinical phenotype at the age of 2 days: bilateral generalized arthrogryposis with predominant bilateral elbow and knee flexion contractures, right foot talipes equinovarus, and left foot vertical talipes. **(B)** Clinical phenotype at the age of 18 months: wide forehead, bitemporal narrowing, hypertelorism, bilateral epicanthus, divergent strabismus, small depressed nasal bridge, small nose, short columella, anteverted nares, smooth and long filtrum, micrognathia, myopathic face appearance with facial muscle weakness, wide and opened mouth with an everted lower lip, high arched palate, small and anterior rotated ears with smooth helix and antihelix, and anterior and uplifted ear lobules. **(C)** Clinical phenotype at the age of 7 years and 10 months: myopathic facies with the same previous particularities, high-arched palate, overcrowded teeth, abnormal dental eruption, generalized thin build, hyperlordotic spine, mild elbow and knee contraction bilateral with hypotrophy of calf muscles, more marked on the left side, and overlapping second and third toes bilaterally. **(D)** MRI lower-limb (plain) (R-right, L-left): T1 axial of the calves showing mild atrophy of right gastrocnemius (c) and right soleus (d) muscles and subtle fatty infiltration; moderate atrophy of left anterior tibialis (a), posterior tibialis (b), gastrocnemius (c), and peroneal (e) muscles with prominent fatty infiltration; with a relative increase of the subcutaneous fat thickness in the left calf compared to the right one.

Patient 1, a boy aged 7 years and 10 months is the first child of a healthy and consanguineous Arab couple (first-degree paternal cousins) (Figure 1). He was born by natural delivery at 40 gestational weeks after a gestation with polyhydramnios, decreased, and weak fetal movements. His birth weight was 2.8 kg (15th percentile). Bilateral generalized arthrogryposis with elbow and knee flexion contractures, right foot talipes equinovarus, left foot vertical talipes, inguinal hernia, bilateral cryptorchidism, and hypotonia were observed at birth (Figure 2A). A brain magnetic resonance imaging (MRI) scan performed at 6 months of age was normal. His infancy was marked by poor feeding, food-swallowing difficulties, and recurrent respiratory infections. An early intensive physiotherapy program reduced upper limb arthrogryposis. The lower limb arthrogryposis and talipes required serial casts and surgical interventions that allowed him to walk independently at the age of 20 months.

When seen in the genetic clinic at 18 months of age, the child’s growth parameters were: weight, 9 kg (3rd percentile); height, 81 cm (15th percentile), and head circumference, 46 cm (15th percentile). Particular dysmorphic craniofacial features were observed (Figure 2B), which included a wide forehead, bitemporal narrowing, hypertelorism, bilateral epicanthus, divergent strabismus, a small depressed nasal bridge, a small nose, a short columella, anteverted nares, a smooth and long philtrum, micrognathia, a myopathic facial appearance with facial muscle weakness, a wide and opened mouth with an everted lower lip, a high arched palate, small and anteriorly rotated ears with a smooth helix and antihelix, anterior and uplifted ear lobules, generalized muscle weakness, dropped shoulders, overlapping second and third toes bilaterally, dysphagia, generalized hypotonia, and motor and speech delays.

A Cytoscan HD SNP array (Affymetrix/Genome Diagnostics, Nijmegen, the Netherlands) reported a normal result of arr (1-22) x2, (XY) x1 but detected larger homozygous regions of 83.9 Mb (3% from the autosomal genome). The fukutin gene (FKTN) was suspected to be responsible for the phenotype, but its sequencing was normal. WES (targeted nucleotides covered ≥20X, 99.9%, Genome Diagnostics, Nijmegen, the Netherlands) reported a homozygous missense variant of uncertain significance in *NEB*, NM_001271208.1:c.11969A>C (p.(Asp3990Ala)), and a pathogenic heterozygous missense mutation in the cyclic adenosine monophosphate response element binding protein (*CREBBP*) gene, NM_004380.2:c.5600G>A (p.(Arg1867Gln). Both parents were confirmed as carriers of the *NEB* gene variant, while the *CREBBP* gene variant was identified in the healthy father.

Nemaline myopathy type 2 (NEM2) was considered as the diagnosis. A muscle biopsy was refused by the parents.

Patient 1 was fully able to speak by the age of 3 years and his muscle weakness improved under physiotherapy. His creatine kinase (CK) values also remained normal at serial re-evaluations. At the last visit to the genetic clinic at 7 years and 10 months of age (Figure 2C), his weight was 18 kg (1^st^ percentile), his height was 117 cm (5th percentile), and his head circumference was 50 cm (5th percentile). The same particular myopathic facies with overcrowded teeth was noted again. His dysphagia had improved after intense occupational therapy. He showed winged scapulae bilaterally, a hyperlordotic spine, mild elbow and knee contraction bilaterally, and a generalized thin build with hypotrophy of the calf muscles, more marked on the left side. He was able to run but suffered frequent falls. Lower-limb muscle MRI performed at 7 years and 10 months of age revealed normal thigh muscles but moderate atrophy and fatty infiltration of the anterior, lateral, and posterior lower leg muscles. The left anterior and posterior tibialis, peroneal and gastrocnemius muscles showed predominant atrophy, with a relative increase in subcutaneous fat thickness (Figure 2D). Electrocardiography reported bradycardia, but a heart ultrasound was normal. Ophthalmology follow-up reported +3.5 D for both eyes, with divergent strabismus. He showed normal cognitive development and got good grades in school.

### Case 2

**FIGURE 3:**
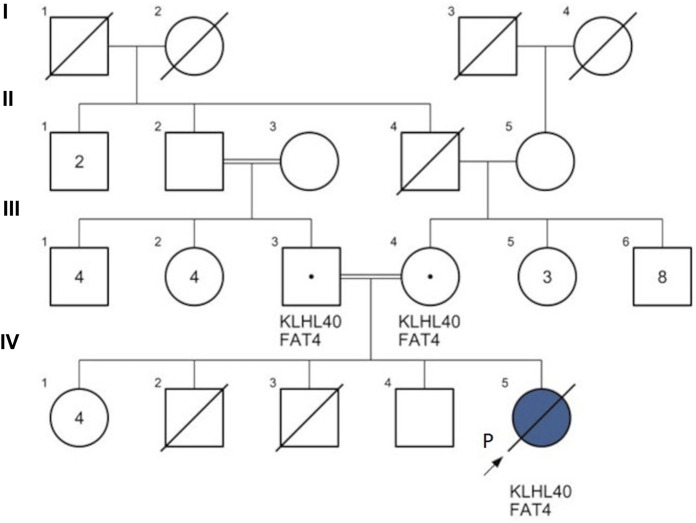
Family pedigree of patient 2. KLHL40: Kelch-like family member 40 gene variant c.1405G>A; FAT4: FAT atypical cadherin 4 gene variant c.13178A>T. Proband (P) IV5 (blue): patient 2 was diagnosed with nemaline myopathy type 8 (KLHL40 gene homozygous c.1405G>A), FAT4 gene homozygous c.13178A>T. The couple II-2 & II-3 are 2nd-degree relatives. IV-2: a full-term neonatal death after 24 h, unknown cause; IV-3: a 2nd-trimester intrauterine fetal death (IUFD) at 16 gestational weeks, unknown cause.

Patient 2 was a 10-week-old girl and the sixth child of a healthy, consanguineous Arab couple (first-degree paternal cousins) who had previously lost a pregnancy by second-trimester intrauterine fetal death and a child with severe congenital hypotonia and arthrogryposis by early neonatal death (Figure 3). The patient was born by natural delivery at 37 weeks of gestation after a pregnancy with significant polyhydramnios and decreased fetal movements. At birth her weight was 2.67 kg (30th percentile), her length was 47 cm (35th percentile), and her head circumference was 37 cm (99th percentile). She was apneic and bradycardic at birth and required active resuscitation, tracheal intubation, and neonatal intensive care unit admission. Her APGAR scores were 3, 4, and 5 points at 1, 5, and 10 min, respectively. On examination, her temperature was 36.3°C, her heart rate was 162 bpm, her respiratory rate was 52 breaths/min, and her oxygen saturation was 96% with FiO_2_ of 40%.

**FIGURE 4:**
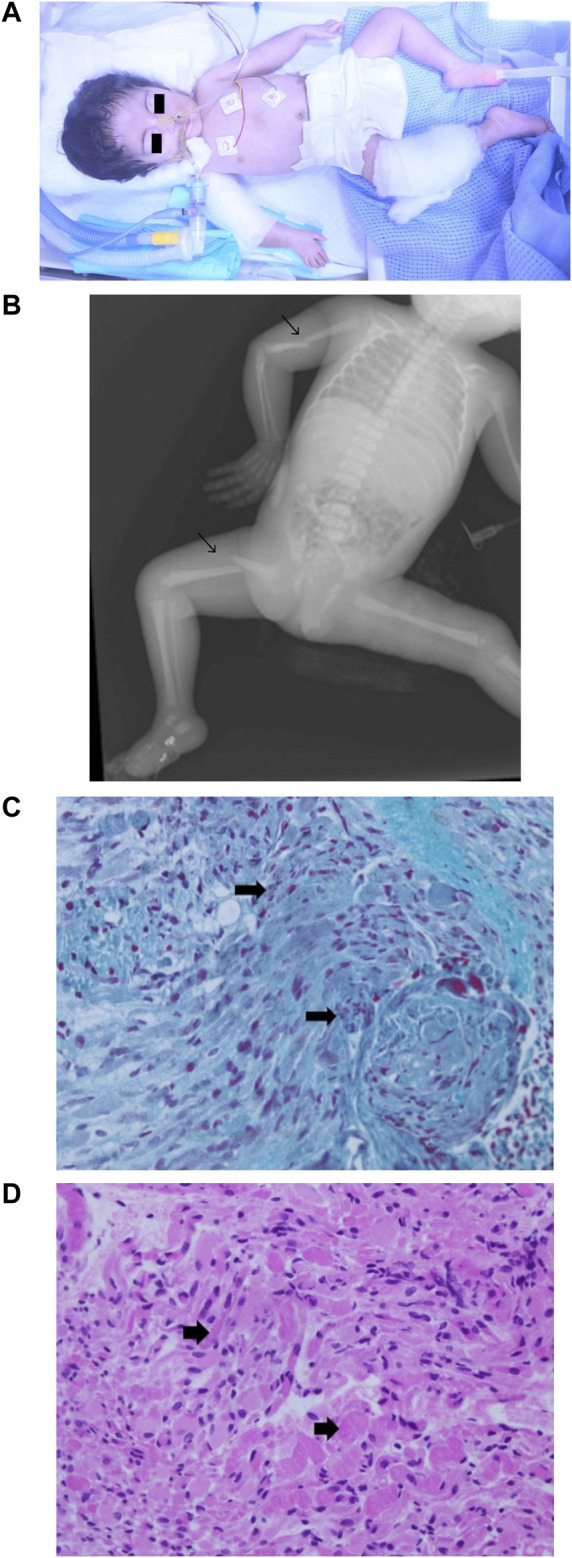
Phenotype of patient 2. **(A)** Clinical phenotype at the age of 1 day: craniofacial dysmorphism with narrowing of the bitemporal diameter, myopathic facies, exophthalmia, bilateral epicanthal fold, wide and depressed nasal bridge, short nose, vertical nostrils, long smooth philtrum, thin lips, micrognathia, microtia, short neck, small chest and rib cage, arthrogryposis with joint stiffness of the upper and lower limbs, right wrist with ulnar deviation, flexion of the left fingers, marked hip abduction, and talipes equinovarus. **(B)** X-ray skeletal survey: right mid-shaft humeral and right proximal femoral dislocated non-communicated fractures. **(C)** Muscle biopsy (left thigh) hematoxylin and eosin staining 40X: variation in muscle fiber size with degenerative changes. **(D)** Muscle biopsy (left thigh) Gomori trichrome stain 40X: degenerative muscle fibers containing red to purple rod inclusions in sarcoplasm.

Physical examinations revealed severe hypotonia and diminished deep tendon reflexes (Figure 4A). Multiple congenital anomalies were observed, including craniofacial dysmorphism with narrowing of the bitemporal diameter, myopathic facies, exophthalmia, a bilateral sluggish pupillary reflex with bulbar muscle weakness, a bilateral epicanthal fold, a wide and depressed nasal bridge, a short nose, vertical nostrils, a long and smooth philtrum, thin lips, micrognathia, microtia, a short neck, a small chest and rib cage, arthrogryposis with joint stiffness of the upper and lower limbs, a right wrist with ulnar deviation, flexion of the left fingers, marked hip abduction, talipes equinovarus, right mid-shaft humeral and right proximal femoral dislocated non-communicated fractures (Figure 4B), and hypoplastic genitalia. The baby remained ventilator-dependent because of severe respiratory failure secondary to respiratory muscle weakness. Feeding was done *via* a nasogastric tube as there was no sucking reflex.

The karyotype analysis was normal (46,XX). Other investigations also revealed normal levels of the muscle enzymes (CK, 266 IU/L; CK-MB, 29.4 IU/L) and normal muscle-specific receptor tyrosine kinase levels. At this point, a rare myopathy was suspected. WES (CentoXome Solo NGS-based CNV, targeted nucleotides covered ≥20X, 99.09%, Centogene, Rostock, Germany) reported two different variants of uncertain significance: a *KLHL40* homozygous missense variant, NM_152393.3:c.1405G>A, p.(Gly469Ser), and a fat atypical cadherin 4 (*FAT4*) gene homozygous missense variant, NM_001291303.1:c.13178A>T, p.(Asp4393Val). The parents were confirmed to be double carriers for both genes variants.

Variations in muscle fiber size with degenerative changes were visualized after hematoxylin and eosin staining of a skeletal muscle biopsy sample (Figure 4C). A high-power microscopic view after Gomori trichrome staining revealed red to purple rod inclusions in the sarcoplasm of degenerative muscle fibers (Figure 4D), linking the molecular genetic result to the muscular pathology features of NMs. The patient was diagnosed with nemaline myopathy type 8 (NEM8), developed severe respiratory failure, and passed away at the age of 10 weeks.

## Discussion

NMs usually present as early-onset proximal muscle weakness, and many cases are suspected prenatally. Prenatal ultrasonographic signs of NMs correlate with a poor prognosis and an increased risk of mortality. Fetal akinesia, polyhydramnios, arthrogryposis, and foot deformities are associated with *NEB* and *KLHL40* gene variants (Lawlor, et al., 2011; Feingold-Zadok et al., 2017; Yeung et al., 2020; Yi et al., 2021). Both patients showed reduced fetal movement, polyhydramnios, neonatal arthrogryposis, and talipes, underlining again the importance of ultrasound imaging for the early detection of NMs. The earlier a rare myopathy is diagnosed, the easier it will be to quickly intervene and manage the condition, identify the recurrence risk, and initiate prevention.

The *NEB* gene variant, at exon 80, NM_001271208.1:c.11969A>C (p.(Asp3990Ala)) identified in patient 1 was assessed as a variant of unknown significance that led to the substitution of a strongly conserved nucleotide and amino acid residue. Nebulin has a fundamental role in skeletal muscle and variants affecting highly conserved regions of *NEB* may be regarded as pathogenic. To our knowledge, this specific variant has not been reported before in the literature or the Genome Aggregation Database (gnomAD) (https://gnomad.broadinstitute.org/); it is also not listed in ClinVar (https://www.ncbi.nlm.nih.gov/clinvar/) or Varsome (https://varsome.com/variant/hg38/NEB). It may have a destabilizing effect on the protein, as predicted by Polyphen 2.0, leading to protein dysfunction. This variant is predicted as being deleterious by Combined Annotation Dependent Depletion (CADD), with a scaled score of 28.7 points (https://cadd.gs.washington.edu/snv). Considering the muscular phenotype of polyhydramnios, fetal hypokinesia, arthrogryposis, myopathic facies, neck flexor weakness, a nasal voice, dysarthria, dysphagia, and muscle weakness, we concluded that this homozygous missense *NEB* gene mutation was a likely candidate for the disease-causing variant seen in patient 1.

Classifying patients early based on their clinical phenotype is challenging, but disease progression during childhood enables the severity of their condition to be clarified (Lehtokari et al., 2014). At the age of 7 years and 10 months, patient 1 had good motor milestones and a slowly progressive, more distal lower limb myopathy corresponding to the classical NEM2 type. Lower-leg MRI showed findings already reported in this NEM2 type (Jungbluth et al., 2004; Gurgel-Giannetti et al., 2022); notably, the myopathy had completely spared the boy’s thigh muscles and predominantly led to atrophy of the left posterior and lateral calf muscles.

Nebulin is also present in the heart and brain, and cardiomyopathy is reported in about 13% of NEM2 patients (Amburgey et al., 2021). Electrocardiography showed bradycardia, but the cardiac ultrasound was reported to be normal for patient 1.

More than 200 *NEB* pathogenic variants are associated with a wide spectrum of variable phenotypes, from severe to mild forms, with associated learning and intellectual disabilities (variants in exons 27 and 143) (Jin et al., 2014; Lehtokari et al., 2014; Amburgey et al., 2021). No patients have been reported to have variants in exon 80 of the *NEB* gene. Our patient had a speech delay and mild learning difficulties as a toddler but experienced normal mental development and a slower myopathy progression.

Patient 1 was included in an intensive rehabilitation program and regularly followed up by a multidisciplinary medical team. He has benefited from physiotherapy and speech and behavioral therapies. His good evolution underlines the importance of both genetic diagnosis and early, sustained intervention.

His parents were confirmed carriers of the same *NEB* variant, and genetic counseling helped them to understand their recurrence risk and to make informed reproductive decisions. They opted for *in vitro* fertilization (IVF) and preimplantation genetic testing for the *NEB* gene variant (PGT-M), and their next pregnancy ended at term with two healthy male dizygotic twins. Multiple relatives later requested preconception carrier genetic testing, including for other rare genetic disorders reported in their extended consanguineous family, such as Niemann-Pick disease type 2C (Figure 1).

The KLHL40 gene variant, at exon 3, NM_152393.3:c.1405G>A (p.(Gly469Ser)), identified in our patient 2 led to the substitution of a conserved amino acid residue. This variant was reported before in ClinVar and Varsome as having uncertain significance for NEM8. It was assessed as likely pathological by ACMG, as probably damaging by the *in silico* prediction tool Polyphen 2.0 (https://genetics.bwh.harvard.edu/pph2/) with a score of 0.996 points (sensitivity: 0.55; specificity: 0.98), as disease-causing by Align GVGD (http://agvgd.hci.utah.edu/) and Mutation Taster (www.mutationtaster.org), and has a deleterious scaled CADD score of 31 points. The same variant was reported as a compound heterozygote with a *KLHL40* gene variant c. 1498C>T in two NEM8 patients from Brazil, 17 months and 5 years of age, respectively, with a severe neonatal phenotype but later motor improvement (patients 29 and 30 in the study by Gurgel-Giannetti et al., 2022).

A similar *KLHL40* variant appearing at the same position in exon 3, in a conserved functional domain, but with a different substitution, c.1405G>T, p(Gly469Cys), was found in a Japanese family with NEM8 (family 15 in the study by Ravenscroft et al. (2013)). Molecular modeling assessed this substitution as one that most likely destabilized the hydrophobic core of the kelch domain and reduced protein stability. It is highly probable that the *KLHL40* gene variant, c.1405G>A, identified in our patient had the same impact and, to the best of our knowledge, this is the first time it has been reported in a family of Arab origin. The *KLHL40* gene variant, c.1582G>A, is the common variant reported in Japanese, Kurdish, and Turkish patients with NEM8 (Seferian et al., 2016) but the variant, c.1516A>C, has only been reported in East Asian populations (Yeung et al., 2020). Considering the rarity of these cases, further studies might be needed to identify *KLHL40* gene variants linked to NEM8 in the Arab population.

NEM8 is characterized by severe and distinctive fetal and neonatal features, and by an early death in the first months of life (Ravenscroft et al., 2013), with rare cases surviving into adolescence (Seferian et al., 2016). As previously reported (Kawase et al., 2015; Yi et al., 2021), our patient 2, also had fetal hypokinesia and polyhydramnios, a severe phenotype at birth with respiratory failure, long bone fractures, arthrogryposis, and hypotonia. Despite the early interventions, she passed away at the age of 10 weeks. The severity of patient 2’s clinical phenotype in association with the abnormal myofibrils and rod bodies identified by the muscular biopsy, supported the molecular findings and certified the diagnosis of a rare severe case of NEM8. When accepted by the family, a muscle biopsy can clarify the gene variant's impact on the structure and functionality of muscle fibers (Wang et al., 2020). The homozygous *KLHL40* gene variant, c.1405G>A, may explain the intrauterine fetal death and the other neonatal fetal death previously recorded in this family.

A heterozygous missense variant was identified in exon 31 of the *CREBBP* gene, NM_004380.2:c.5600G>A (p.(Arg1867Gln)), in patient 1. This variant is classified as pathogenic by the American College of Medical Genetics and Genomics (ACMG), with a score of 17 points (https://varsome.com/variant/hg38/rs1131691326) and has a deleterious scaled CADD score of 28.9 points. Variants in exons 30 and 31 of the *CREBBP* gene are associated with Menke-Hennekam syndrome 1 (https://www.omim.org/entry/618332) (Menke et al., 2016; Menke et al., 2018). Two patients with craniofacial dysmorphism, intellectual disability, and the same *CREBBP* variant c.5600G>A, were previously reported, including a six-year-old girl [patient 8 in the study by Menke et al. (2016)] and a 57-year-old man [patient C16 in the study by Menke et al. (2018)]. Both patients had other genomic changes (del6p12.3 and dup9q34.3, respectively) with unclear influences on their phenotype. Some of their facial dysmorphic features were assessed as possibly associated with this *CREBBP* gene variant, including a depressed nasal bridge, a short nose, anteverted nares, a short columella, a long philtrum, and decreased elbow extension, which were present in our patient but were also reported previously in the NEM2 phenotype. Patient 1 had a speech delay but not a developmental delay. Segregation analysis studies were conducted on the parents and other relatives (their siblings and cousins). The patient’s father has the same *CREBBP* variant, but none of the signs reported in the literature; therefore, it remains uncertain whether this is a case of non-penetrance and whether this variant has any influence on our patient’s phenotype.

Patient 2 was reported to also be homozygous for a missense variant of uncertain significance in the *FAT4* gene, c.13178A>T, (p.(Asp4393Val)), which was not reported in gnomAD but has been predicted as damaging by the *in silico* tool, Mutation Taster. Pathogenic variants in the *FAT4* gene are associated with autosomal recessive Van Maldergem syndrome type 2, and Hennekam lymphangiectasia-lymphedema syndrome type 2. Hypotonia, respiratory difficulties, and talipes are also features of Van Maldergem syndrome. A typically periventricular nodular heterotopia linked to this syndrome could not be ruled out; therefore, the impact of this variant on our patient’s phenotype, if any, is uncertain.

The professional care and guidance received led to an excellent collaboration with the families of both patients. Genetic counseling facilitated an understanding of complex genetic pathologies, clarifying the impact of consanguinity and the importance of prevention. The counseling helped both families to cope with this rare disease diagnosis and to make informed decisions going forward, regarding adequate management and future offspring. Getting another healthy pregnancy with the help of IVF and PGT-M for the *NEB* gene variant brought a lot of joy to the first family, and both families have become strong advocates of the importance of preconception genetic counseling in the context of consanguinity and a history of affected children.

## Conclusion

Homozygous variants of the *NEB2* gene, c.11969A>C, and the *KLHL40* gene, c.1405G>A, were correlated with the phenotypes of the two patients presented here. This study enriches our knowledge about the phenotypic spectrum of nemaline myopathy caused by variants in *NEB* and *KLHL40* and highlights the importance of detailed prenatal, neonatal, and infancy assessments of muscular weakness associated with complex systemic features. Variants of uncertain significance in genes associated with nemaline myopathies may also contribute to the phenotype. Early and multidisciplinary intervention can improve the outcome, especially in patients with mild forms of the disease. WES is essential for clarifying complex clinical phenotypes encountered in patients from consanguineous families. Targeted carrier screening of extended family members facilitates accurate genetic counseling and enables genetic prevention.

## Data Availability

The datasets for this article are not publicly available due to concerns regarding participant/patient anonymity. Requests to access the datasets should be directed to the corresponding author.

## Abbreviations

NMs, nemaline myopathies; NEM2, nemaline myopathy type2; NEM8, nemaline myopathy type 8.
